# Supplementary material for: Inverse Design of Amorphous Materials With Targeted Properties
Source: Adv Mater. 2026 Jun 9;38(39):e22493. doi: 10.1002/adma.202522493 (PMC13361175; doi:10.1002/adma.202522493)
Supplement: Supplementary file 1 — Supporting File: adma73640‐sup‐0001‐SuppMat.pdf. [file ADMA-38-e22493-s001.pdf]

Supplemental Information for "Inverse Design of Amorphous Materials with Targeted Properties"

Jonas A. Finkler, Yan Lin, Tao Du, Jilin Hu, Morten M. Smedskjaer

1 Data Set Generation

1.1 Multi Element Glass Data Set

We created the multi element glass (MEG) data set to test our model's performance on data including a larger variety of elements. As also mentioned in the Methods section, the data set consists of 9,027 samples, containing 11 different elements. Initial structures were generated from varying compositions of the glass formers SiO_2 and P_2O_5, and the modifiers Al_2O_3 Li_2O, BeO, K_2O, CaO, TiO_2, BaO and ZnO.

Structural samples and corresponding properties of the MEG data set were obtained using the workflow described below. Simulations were performed using LAMMPS [1] software and the Bertain–Menziani–Pedone (BMP)-shrm potential [2].

1. Elemental compositions were generated to include different ratios of the three glass formers, up to four different modifiers with total concentration of 40 % relative to the glass former concentration.

2. Initial structures of the generated compositions, containing roughly 800 atoms, were created by randomly placing the atoms in a simulation cell with a volume $V = 3\sum_i \frac{4}{3}\pi r_i^3$, with r_i being the covalent radius of atom i. The atoms positions were then adjusted to ensure that no two atoms were closer than the sum of their respective covalent radii. Finally, a local geometry optimization was performed to optimize the atomic positions and cell dimensions.

3. To ensure proper melting while avoiding evaporation, an initial temperature for the melt-quench procedure needed to be determined for each composition. For this task, the initial cells were doubled in size along one dimension to form a vacuum region. A short molecular dynamics (MD) simulation was then performed in the NVT ensemble during which the temperature was

increased up to 8000 K for a duration of 100 ps. The evaporation temperature T_{evap} was then identified at the onset of pressure increase during the dynamics simulation.

4. Structural samples were obtained from a melt-quench simulation in the NPT ensemble, initialized at $T_{\mathrm{init}} = \frac{3}{4}T_{\mathrm{evap}}$. The samples were first melted for 400 ps, then quenched to 300 K at 5 K/ps and finally equilibrated for 300 ps. Out of 9,240 compositions, 213 samples were identified that did not melt properly during the initial phase of the simulation and were thus excluded from the final data set.

5. Melt-quenched samples were then equilibrated at 50 K for 100 ps and subsequently heated to 500 K over 500 ps to extract heat capacities and thermal expansion coefficients.

6. Samples were also relaxed to compute the elasticity tensor using finite differences of the stress tensor.

Due to the finite number of atoms, some amount of uncertainty in the computed properties is expected. To assess these, we performed two independent runs of the workflow for a random subset of compositions, resulting in two sets of structural samples for which properties were calculated. For one set of samples, the final heating simulation of the workflow was then repeated with the same initial structure but using a different random seed to assess the variability introduced by the heating simulation. Correlation plots of all properties and corresponding Pearson correlation coefficients are shown in Fig. S1. The elastic constants, which were deterministically computed from the structural samples, correlate well between the independent runs of the workflow, indicating a strong dependence on the composition. Similarly, the evaporation temperature shows a strong correlation between the independent runs. The thermal expansion coefficient and the molar heat capacity show a weaker correlation between the independent runs but a good agreement between the two heating simulations. Overall, this indicates that the simulation workflows to obtain glass properties work reliably, with variability being attributed to differences between the structures of the samples.

Heat capacities were obtained as the gradient of a linear fit to the total energy versus temperature of the heating simulation in step 5 of the workflow. Similarly, the thermal expansion coefficient (α) at room temperature was obtained from a linear fit $V(T)$ to the volume versus temperature of the heating simulation and calculated as

$$\alpha = \frac{1}{V(T)} \left. \frac{\partial V(T)}{\partial T} \right|_{T=300\,\mathrm{K}} . \tag{1}$$

Elastic constants were obtained as described in Section 3.

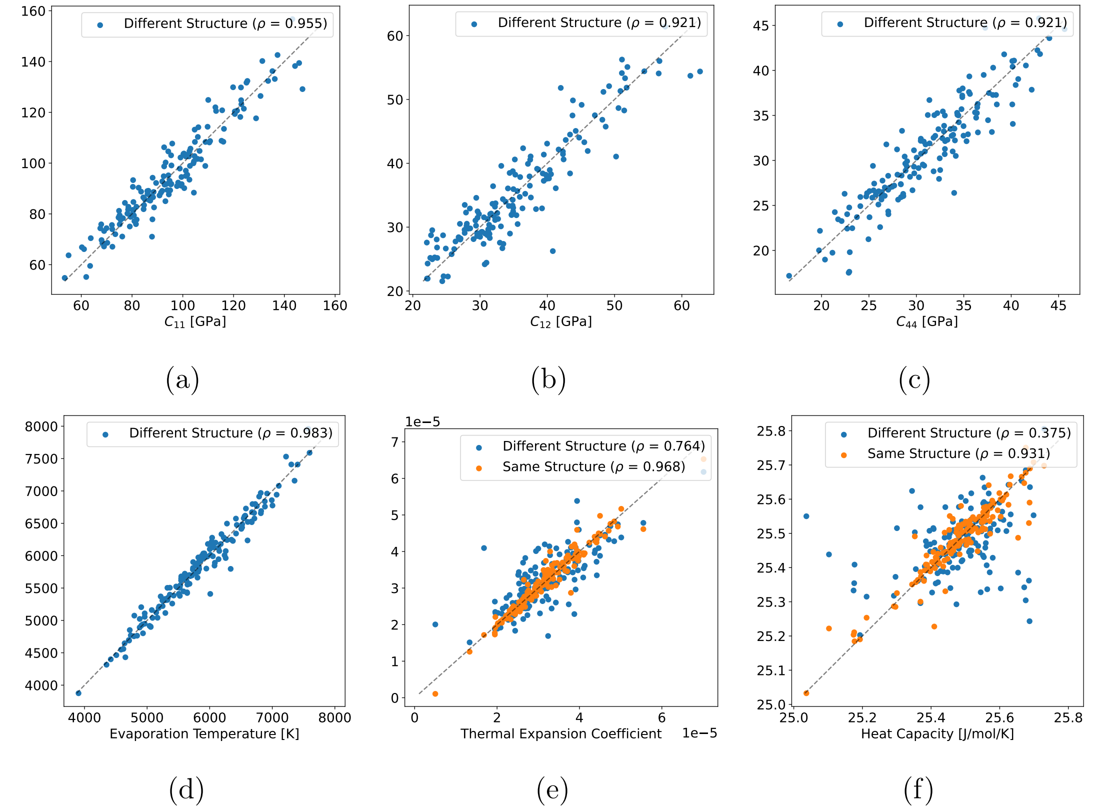

Supplemental Figure S1: Parity plots of the independent elastic constants C_{11} (a), C_{12} (b) and C_{44} (c), the evaporation temperature T_{evap} (d), the thermal expansion coefficient (e), and the molar heat capacity (f) for a random subset of samples of the MEG data set. The properties were computed from two independent runs of the workflow, initialized with the same compositions. Properties obtained from the heating simulation of the workflow were computed for a third time by re-running the heating simulation initialized with the same structural sample but using a different random seed. Pearson correlation coefficients ρ are shown in the figure legends.

1.2 Amorphous Silica Data Set

We developed the amorphous silica data set to investigate our model's performance acting exclusively on the structure of the sample without changing the material's composition. As such, all $6,000$ samples share the composition of pure silica, SiO_2. To maximize the variation of properties between the samples generated with the same simulation workflow, relatively small unit cells were chosen with the number of atoms uniformly selected in the range of 80 to 250. Atoms were initially placed in a unit cell with a volume $V = 4\sum_i \frac{4}{3}\pi r_i^3$, with r_i being the

covalent radius of the i-th atom, avoiding unphysical overlap between neighboring atoms. A local structure relaxation was performed on the initial configuration followed by an MD simulation in the NPT ensemble at 3500 K for 2000 ps. To limit the effects of relaxation, which we observed for our other data sets, we used an instantaneous quenching procedure by performing a local structure optimization and a subsequent equilibration at 300 K for 10 ps. Elastic constants were computed from the relaxed final structures using finite differences of the stress tensor as described in Section 3. All simulations were performed using LAMMPS [1] software and the Tersoff potential parameterized by Munetoh *et al.* [3].

1.3 Amorphous Silicon Data Sets

We created three data sets (*melt*, *quench* and *anneal*) of amorphous silicon to study the effects of relaxation on the generation performance. All three data sets were created using LAMMPS [1] software with the Stillinger–Weber potential [4] and consisted of 10 000 samples each. The simulations were initialized with a unit cell containing 256 atoms of crystalline silicon, but different thermal schedules were applied to obtain the final samples. All MD simulations were performed in the NPT ensemble at zero pressure.

The *melt* data set was generated by heating the crystalline silicon from 2500 K to 3000 K over 200 ps, equilibrating the melt for 300 ps, and then cooling it down again to 2500 K at a rate of 10^{12} K/s. The final samples were taken after equilibrating for another 300 ps at 2500 K.

The *anneal* and *quench* data sets were both initialized at 300 K, heated to 2500 K over 200 ps, and equilibrated for 300 ps. Samples for the *anneal* data set were then cooled down at a rate of 10^{12} K/s to 300 K and equilibrated for another 300 ps, while the cooling step was omitted for samples for the *quench* data set. The structures of the *anneal* data set were thus allowed to relax during the cooling period, while the *quench* samples were obtained from an almost instantaneous quenching procedure. However, a small amount of relaxation is still expected during the time period taken by the thermostat to adjust the temperature of the system to the lower target value.

Ring sizes were computed according to the definition of Guttman [5] and reported as the number of Si atoms in the ring. Atoms where considered bonded if the distance between them was below the sum of their covalent radii ($r_{\mathrm{Si}} = 1.11$ Å, $r_{\mathrm{O}} = 0.66$ Å) multiplied with a factor of 1.3.

Since ghost atoms were used for the generation of the reported SiO_2 structures, stoichiometric balance of the generated samples is not strictly guaranteed. Instead, the model learns to predict the correct ratio between Si and O atoms in the samples. Histograms of the Si to O_2 ratios in the generated samples are shown in Fig. S2. We note that the figures included in the main text only contain perfectly

stoichiometrically balanced samples. Parity plots of the shear modulus and the average ring size, including also the non-stoichiometric samples, are shown in Fig. S3. The dependence of the valid fraction on the target property value is shown in Fig. S9.

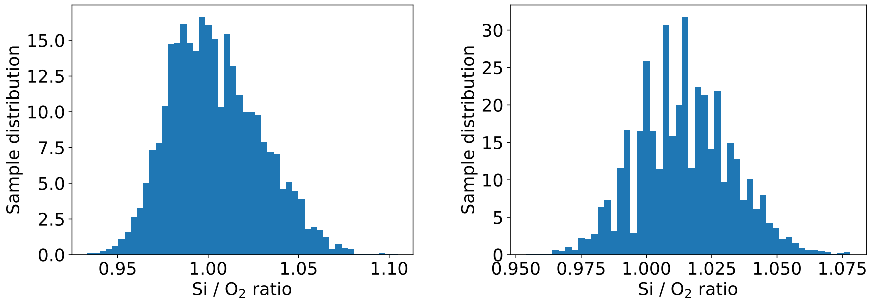

(a) (b)

Supplemental Figure S2: Histograms of the ratio between Si and O_2 content in the samples generated by AMDEN conditioned on shear modulus (a) and average ring size (b).

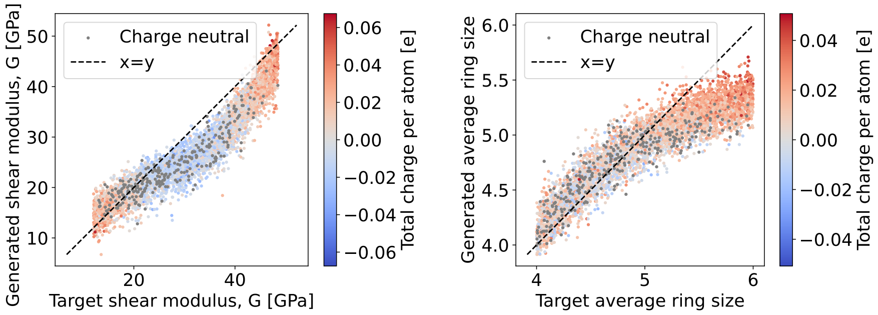

(a) (b)

Supplemental Figure S3: Parity plots between target and generated shear modulus (a) and average ring size (b). The total system charge per atom is indicated by color and was calculated using formal charges of $+2\,e$ and $-1\,e$ for Si and O atoms, respectively.

2 Structural features of the amorphous Si data set

To analyze the quality of the structures generated by AMDEN, we computed radial distribution functions, bond angle distributions, structure factors, the potential

energy distribution, coordination number distributions, and Voronoi volume distributions of the generated and the training samples. Bond angle distributions were computed using a radial cutoff of 2.7 Å. All features were computed before and after performing a local geometry optimization of the structures. Features obtained from the standard denoising procedure are shown in Fig. S4, while those obtained from the Hamiltonian Monte Carlo (HMC) denoising procedure are shown in Fig. S5. Figures shown in the main text are included here again for completeness.

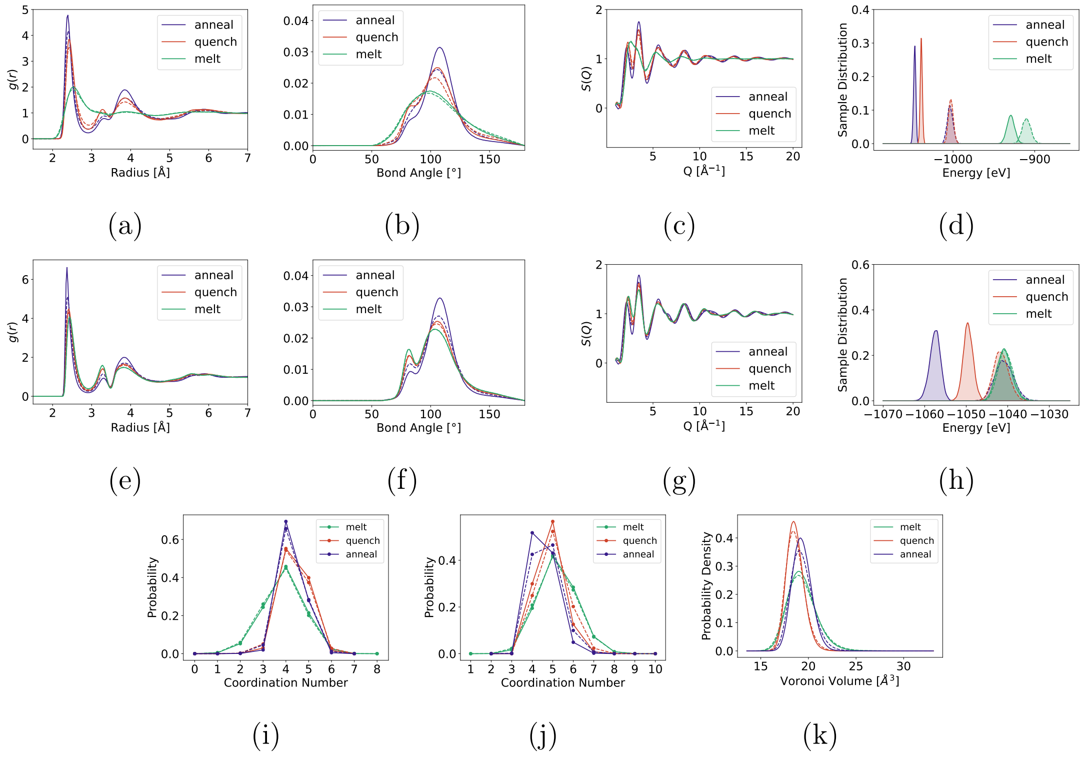

Supplemental Figure S4: Radial distribution function (a), bond angle distribution (b), structure factor (c), energies (d), coordination number distributions at cutoff radii of 2.8 Å (i) and 3.0 Å (j), and Voronoi volume distribution (k) of the generated structures compared to the training data. Panels (e), (f), (g) and (h) show the features in the same order after performing a local geometry optimization using the Tersoff potential used for generating the training data. Training data are shown by solid lines, while dashed lines are obtained from the AMDEN-generated samples.

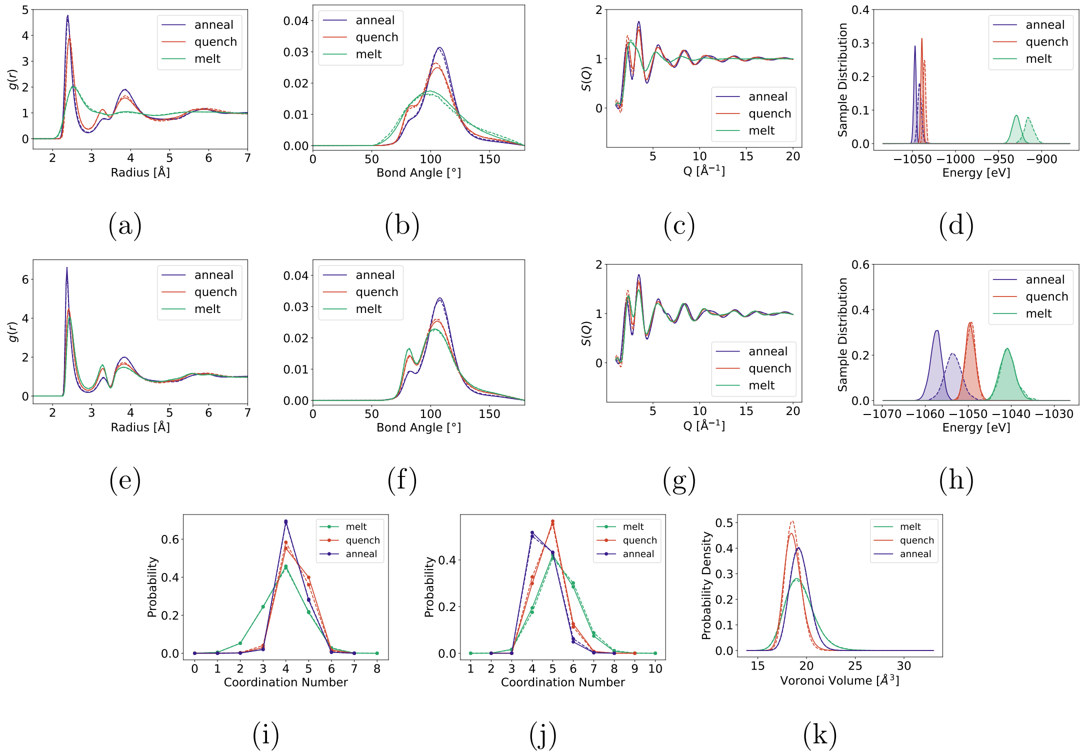

Supplemental Figure S5: Radial distribution function (a), bond angle distribution (b), structure factor (c), energies (d), coordination number distributions at cutoff radii of 2.8 Å (i) and 3.0 Å (j), and Voronoi volume distribution (k) of the structures generated using Hamiltonian Monte Carlo (HMC) denoising compared to the training data. Panels (e), (f), (g) and (h) show the features in the same order after performing a local geometry optimization using the Tersoff potential used for generating the training data. Training data are shown by solid lines, while dashed lines are obtained from the AMDEN-generated samples.

3 Mechanical properties

Young's and shear moduli were computed using the strain tensor C_{ijkl}. First, a local geometry optimization was performed on the structural samples to obtain the relaxed atomic positions and lattice vectors. The strain tensor was calculated as the derivative of the stress tensor σ_{ij} with respect to the strain ε_{kl}, i.e.,

$$C_{ijkl} = \left. \frac{\partial \sigma_{ij}}{\partial \varepsilon_{kl}} \right|_{\varepsilon=0} . \tag{2}$$

Finite differences were used to calculate the derivatives and atomic positions were relaxed after straining the unit cell before the stress tensor was computed.

Since the investigated samples are largely isotropic, we can reduce C_{ijkl} to C_{ij} using Voigt notation, averaging redundant entries in the full tensor. The Young's and shear moduli are then computed as

$$E = \frac{(C_{11} - C_{12}) \cdot (C_{11} + 2\,C_{12})}{C_{11} + C_{12}} \tag{3}$$

and

$$G = C_{44} \tag{4}$$

respectively [6].

4 Structural features of the MEG dataset

To validate the local structure of the generated multi-element glass (MEG) samples, we computed partial radial distribution functions (RDFs) and cumulative coordination numbers for six element–oxygen pairs: Si–O, P–O, Al–O, Li–O, Ti–O, and Ca–O. These include all three network formers (Si, P, Al) and the three most abundant modifier cations (Li, Ti, Ca), which together account for the majority of cation sites in the dataset. The remaining modifiers (K, Ba, Be, Zn) are too sparse for statistically reliable comparison. The generated samples are taken from the Li-conditioned inverse design runs (targeting a Li molar fraction of 0.15 and Young's modulus in the range 20–160 GPa), which produce structures with compositions that differ from the average training distribution.

Supplemental Figure S6 shows the partial RDFs $g(r)$ comparing the training data with generated samples (with and without HMC refinement). Standard denoising reproduces the correct first-shell peak positions for all six pairs, but the peaks are systematically lower and broader than the training data, indicating incomplete relaxation of the local bonding environments. HMC refinement substantially improves the agreement, sharpening the first-shell peaks to closely match the training data. This is consistent with the improvements observed for the amorphous Si anneal dataset. The Li–O pair is an exception: the HMC-refined samples show a slightly higher first peak than the training average, which reflects the elevated Li content in the conditioned generation.

Supplemental Figure S7 presents the cumulative coordination number $n(r)$, which counts the average number of oxygen neighbors within distance r for each cation type. The first-shell plateau values confirm that the generated structures reproduce the expected coordination numbers: $\sim$4 for Si, P, and Al, and higher values for the modifier cations. The agreement is strongest in the first coordination shell, where the HMC-refined curves closely track the training data, while the standard denoising curves show small but visible offsets. Beyond the first shell,

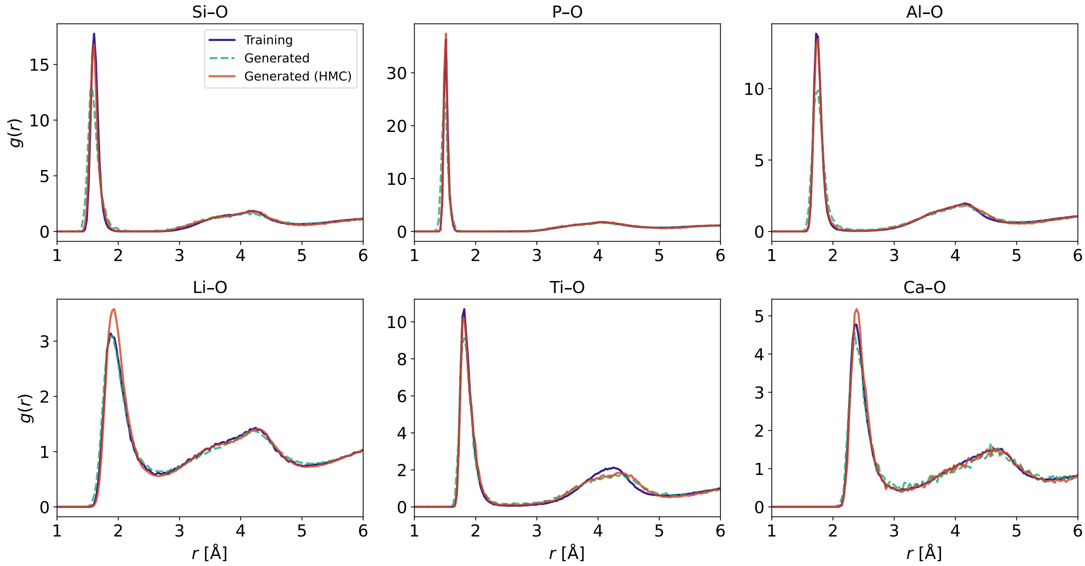

Supplemental Figure S6: Partial radial distribution functions $g(r)$ for six element–oxygen pairs in the MEG system, comparing training data (solid blue), generated samples without HMC denoising (dashed green), and generated samples with HMC denoising (solid red).

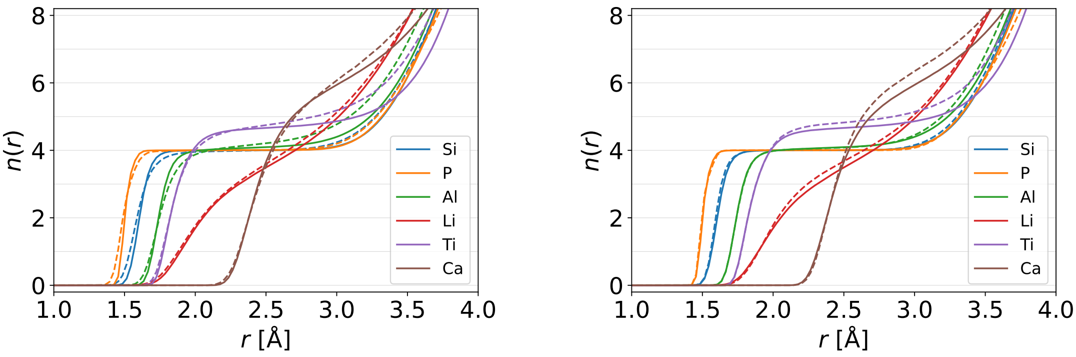

(a) (b)

Supplemental Figure S7: Cumulative coordination number $n(r)$ of oxygen neighbors around each cation type, comparing training data (solid) with generated samples (dashed). (a) Standard denoising. (b) HMC denoising.

minor deviations appear, reflecting the compositional differences between the conditioned generation and the training distribution.

5 Property distributions of generated samples

Supplemental Figure S8 compares the distributions of target properties between the training data and the generated samples for both the MEG and amorphous SiO_2 datasets. In all cases, the generated distributions extend beyond the training range, particularly toward lower property values.

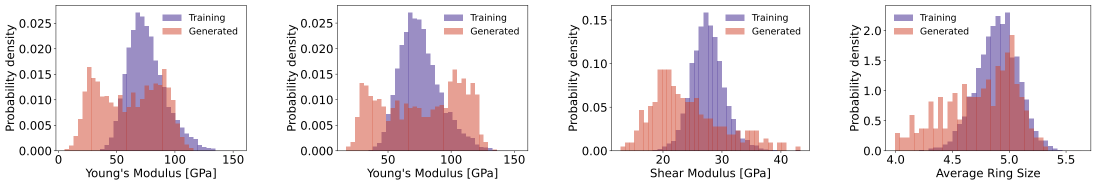

(a) (b) (c) (d)

Supplemental Figure S8: Comparison of property distributions between training data and generated samples. (a) Young's modulus on the MEG dataset with standard denoising. (b) Young's modulus on the MEG dataset with HMC denoising. (c) Shear modulus on the amorphous SiO_2 dataset. (d) Average ring size on the amorphous SiO_2 dataset. In all panels, the generated distributions extend beyond the training range, particularly toward lower property values, indicating extrapolation capability of the model.

6 Generation validity statistics

Since AMDEN uses ghost atoms to control the density of generated structures, stoichiometric balance is not strictly guaranteed. A generated sample is considered valid if its total formal charge is exactly zero, corresponding to an exact Si:O = 1:2 ratio for SiO_2 and the appropriate cation-to-anion balance for the MEG system. For the single-element amorphous Si datasets, all generated samples are valid by construction. Table S1 summarizes the generation validity statistics across all datasets.

For the SiO_2 dataset, the valid fraction is 5.5% (275/5 000) and 6.4% (320/5 000) for the shear modulus and average ring size conditioning, respectively. As shown in Fig. S9, the valid fraction depends on the target property value: it is highest within the training range and drops when the target falls outside the training distribution. No samples fail during generation or property evaluation for either conditioning target.

For the MEG dataset, about 1–2% of generated samples are strictly stoichiometrically balanced (Fig. S10a), reflecting the difficulty of achieving exact charge

neutrality across 11 elements. The number of HMC samples is smaller than for standard denoising due to the higher per-sample computational cost of HMC on this large system (~800 atoms per sample), which also limits the number of stoichiometrically balanced HMC samples. As discussed in the main text, the MEG results are not filtered by stoichiometric balance. Among the MEG samples, 85.7% (2 090/2 440) and 92.6% (910/983) are successfully requenched for standard and HMC denoising, respectively. As shown in Fig. S10b, the requench failures are concentrated in the extrapolative low-E regime: for standard denoising, only 28.5% of samples targeting $E \approx 30\,\mathrm{GPa}$ are successfully requenched, compared to >99% for $E > 70\,\mathrm{GPa}$. HMC denoising substantially improves the requench success rate in the extrapolative low-E regime (60.4% vs. 28.5% at $E \approx 30\,\mathrm{GPa}$), while both methods achieve near-perfect success rates for targets within the training range.

Supplemental Table S1: Generation validity statistics across all datasets. A sample is considered valid if its total formal charge is exactly zero. For amorphous Si, all samples are valid by construction.

Dataset	Denoising	Conditioning	Total	Valid	Valid (%)
a-Si	–	–	–	–	100.0
SiO_2	Standard	G	5 000	275	5.5
SiO_2	Standard	Ring size	5 000	320	6.4
MEG	Standard	E, C_{Li}	2 440	33	1.4
MEG	HMC	E, C_{Li}	983	21	2.1

7 Structural validation of generated SiO_2 samples

To verify that the larger generated SiO_2 cells used for inverse design (350–500 atoms, larger than the training range of 80–250 atoms) remain physically realistic at the medium-range level, we performed independent reference melt-quench MD simulations and compared the resulting ring size distributions.

The reference simulations were performed using the LAMMPS software [1] with the BKS interatomic potential [7], which is widely used for amorphous SiO_2 and is independent of the Tersoff potential [3] used for the training data. For each conditioning target (G and average ring size), the AMDEN-generated cells were melted in the NPT ensemble at 5 000 K for 500 ps, cooled to 300 K at a rate of 1 K/ps, and finally equilibrated at 300 K and zero pressure for 100 ps to obtain the reference glass configurations. The high-temperature melting step erases the initial atomic positions, so the resulting structures represent independent melt-quench references at the same enlarged cell size and composition. Ring size distributions

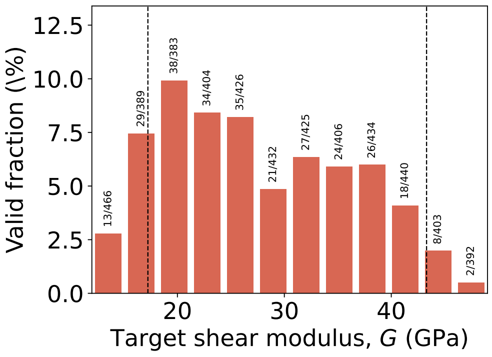

(a)

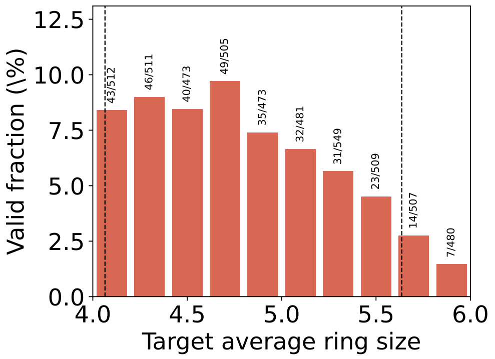

(b)

Supplemental Figure S9: Valid (stoichiometrically balanced) fraction of generated SiO_2 samples as a function of target (a) shear modulus and (b) average ring size. Dashed vertical lines indicate the training data range. Each bar is annotated with the number of valid samples over the total in that bin.

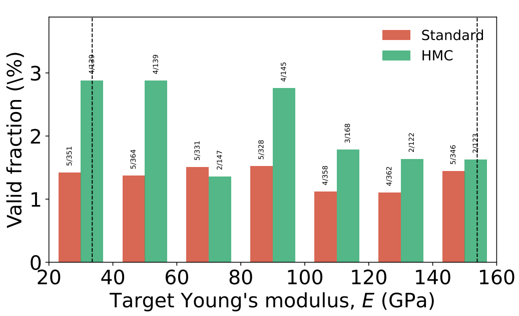

(a)

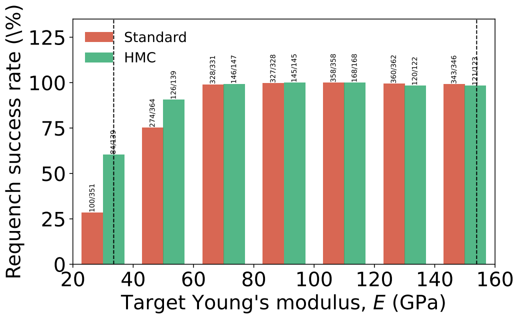

(b)

Supplemental Figure S10: MEG generation statistics as a function of target Young's modulus. (a) Valid (stoichiometrically balanced) fraction. (b) Requench success rate. Dashed vertical lines indicate the training data range. Each bar is annotated with the number of successful samples over the total in that bin.

for both the AMDEN-generated and reference structures were computed according to the Guttman criterion [5].

Supplemental Figure S11 compares the ring size distributions for both conditioning targets. The two distributions agree on the peak location and overall range of ring sizes (3 to 7, concentrated around 4 to 6), consistent with established structural features of amorphous SiO_2, indicating that the larger generated cells remain physically realistic at the medium-range level. Small but systematic differ-

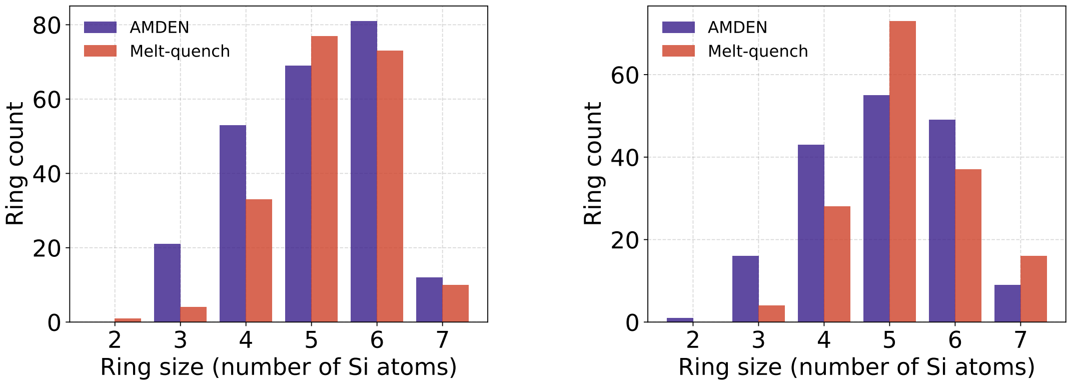

(a) (b)

Supplemental Figure S11: Ring size distributions of AMDEN-generated SiO_2 samples (blue) compared with reference melt-quench MD simulations using the BKS potential (red). (a) Generation conditioned on shear modulus G. (b) Generation conditioned on average ring size. Ring sizes are reported as the number of Si atoms in the ring, computed using the Guttman criterion.

ences are visible: the AMDEN distributions place more weight on smaller (3- and 4-membered) rings, reflecting the structural variation that AMDEN produces in response to the conditioning target. This effect is most pronounced in the ring-size-conditioned case (Fig. S11b), where AMDEN shifts probability mass toward smaller rings to match the lower target ring sizes. Together with the target–property parity already shown in Fig. 4 of the main text, these results indicate that AMDEN can bias the medium-range network topology in directions that are not naturally accessible to the melt-quench procedure while still producing physically realistic structures.

To check that this comparison is not dominated by finite-size effects, we repeated the same melt-quench protocol with the Tersoff potential [3] (the same potential used to generate the training data) at three cubic supercells of the base SiO_2 cell ($1\times1\times1$, $2\times2\times2$, $3\times3\times3$), spanning the range of AMDEN-generated cell sizes (350–500 atoms). Supplemental Figure S12 overlays the ring fraction distributions of the AMDEN-generated samples for both conditioning targets on the three reference distributions. The three reference distributions are nearly superimposed across the populated 3- to 7-membered ring range, indicating that the reference ring statistics are converged with respect to cell size at the AMDEN scale. The AMDEN distributions for both conditioning targets track the reference closely at the peak (5-membered rings) and across sizes 4 to 7, while displaying the same modest shift toward smaller (3- and 4-membered) rings already seen in Fig. S11. Two conclusions

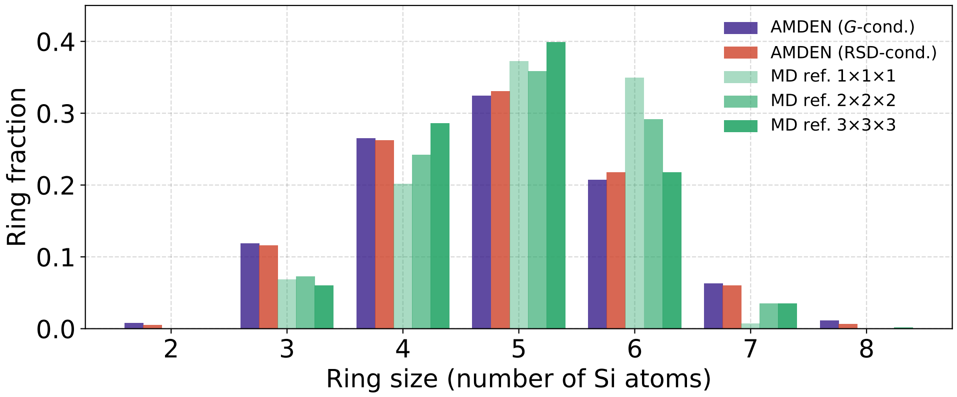

Supplemental Figure S12: Ring fraction distributions of AMDEN-generated SiO_2 samples for both conditioning targets (blue: G-conditioned; red: ring-size-conditioned), overlaid on reference Tersoff melt-quench MD simulations at three cubic supercell sizes (1×1×1, 2×2×2, 3×3×3). Ring sizes are reported as the number of Si atoms in the ring, computed using the Guttman criterion. The reference distributions are stable across cell sizes, and the AMDEN distributions track them across the populated range with the same small shift toward smaller rings observed in Fig. S11.

follow. First, the medium-range agreement reported above persists across roughly an order of magnitude in cell volume and is therefore not an artifact of cell size. Second, the shift toward smaller rings reflects a structural bias introduced by AMDEN's conditioning rather than a finite-size effect, reinforcing the interpretation that AMDEN can access medium-range topologies that the melt-quench procedure does not naturally produce.

References

[1] A. P. Thompson, H. M. Aktulga, R. Berger, D. S. Bolintineanu, W. M. Brown, P. S. Crozier, P. J. in 't Veld, A. Kohlmeyer, S. G. Moore, T. D. Nguyen, R. Shan, M. J. Stevens, J. Tranchida, C. Trott, and S. J. Plimpton, Comp. Phys. Comm. **271**, 108171 (2022).

[2] M. Bertani, M. C. Menziani, and A. Pedone, Phys. Rev. Mater. **5**, 045602 (2021).

[3] S. Munetoh, T. Motooka, K. Moriguchi, and A. Shintani, Comput. Mater. Sci. **39**, 334 (2007).

[4] F. H. Stillinger and T. A. Weber, Phys. Rev. B **31**, 5262 (1985).

[5] L. Guttman, J. Non-Cryst. Solids **116**, 145 (1990).

[6] J. I. Gersten and F. W. Smith, *The Physics and Chemistry of Materials* (John Wiley & Sons, Inc., New York, Chichester, Weinheim, Brisbane, Singapore, Toronto, 2001) a Wiley-Interscience publication. Joel I. Gersten and Frederick W. Smith, The City College of the City University of New York.

[7] B. W. H. van Beest, G. J. Kramer, and R. A. van Santen, Phys. Rev. Lett. **64**, 1955 (1990).
